# A Novel Brucine Gel Transdermal Delivery System Designed for Anti-Inflammatory and Analgesic Activities

**DOI:** 10.3390/ijms18040757

**Published:** 2017-04-03

**Authors:** Ping Wu, Qin Liang, Pei Feng, Chunyan Li, Chunguang Yang, Hongsuo Liang, Huaibo Tang, Cijun Shuai

**Affiliations:** 1 School of Chemistry, Xiangtan University, Xiangtan 411105, China; pingwu@xtu.edu.cn (P.W.); liangqin@hotmail.com (Q.L.); lichunyan@xtu.edu.cn (C.L.); yangchunguang2016@hotmail.com (C.Y.); 2 Hunan Province Engineering Research Center of Bioactive Substance Discovery of Chinese Medicine, School of Pharmacy, Hunan University of Chinese Medicine, Changsha 410208, China; 3 Key Lab of Environment-Friendly Chemistry and Application in Ministry of Education, Xiangtan University, Xiangtan 411105, China; 4 State Key Laboratory of High Performance Complex Manufacturing, Central South University, Changsha 410083, China; fengpei@csu.edu.cn; 5 Joint Surgery Department of the Second People’s Hospital of Nanning City, Guangxi Zhuang Autonomous Region, Nanning 530021, China; lianghongsuo@hotmail.com

**Keywords:** brucine gel, anti-inflammatory, analgesic activities, transdermal delivery

## Abstract

The seeds of *Strychnos nux-vomica* L., as a traditional Chinese medicine, have good anti-inflammatory and analgesic activities. However, it usually leads to gastrointestinal irritation and systemic toxicity via oral administration. In the study, it was discovered that a novel gel transdermal delivery system contained brucine, the main effective component extracted from *Strychnos nux-vomica*. Results showed that the brucine gel system inhibited arthritis symptoms and the proliferation of the synoviocytes in the rat adjuvant arthritis model, which indicated its curative effect for rheumatoid arthritis. Meanwhile, it significantly relieved the xylene-induced ear edema in the mouse ear swelling test, which manifested its anti-inflammatory property. Moreover, the brucine gel eased the pain of paw formalin injection in the formalin test, which demonstrated its analgesic effects. In addition, the brucine significantly inhibited lipopolysaccharide (LPS)-induced Prostaglandin E2 (PGE2) production without affecting the viability of cell in vitro anti-inflammatory test, which proved that its anti-inflammatory and analgesic actions were related to inhibition of prostaglandin synthesis. It is suggested that the brucine gel is a promising vehicle for transdermal delivery on the treatment of inflammatory disease.

## 1. Introduction

*Strychnos nux-vomica*, a seed of *Strychnos nux-vomica* L. (Loganiaceae), can effectively ease pain and diminish inflammation. Thus, it has been widely used in the treatment of rheumatism and related inflammatory diseases by oral administration in traditional Chinese medicine [1,2,3,4,5]. For instance, it is a major ingredient in many finished herbal products, including “maqian-zi-san”, “shu-feng-ding-tong-pian”, and “fei-bu-wan” [6]. However, it usually leads to violent convulsion, great rise of blood pressure and even lethal poisoning at too high a dosage via oral administration [7]. In fact, its oral therapeutic dosage is very close to the toxic dosage [8]. A novel steady and continuous administration should be designed to avoid oral toxicity.

Transdermal drug delivery system is an administration method through skin absorption, which can continuously deliver drugs and ensure steady plasma drug concentration [9]. Compared with oral administration, this method can avoid gastrointestinal irritation and toxic reaction under the condition of efficacy ensurance [10]. It is generally believed that an appropriate drug for transdermal drug delivery system should have a small molecular weight (<500 Da), high purity and good lipophilicity. In this sense, *Strychnos nux-vomica* composed of many components of plants is not suitable for transdermal delivery [11].

Brucine is a major effective component of *Strychnos nux-vomica* [10,11,12]. Significantly, it has a small molecular weight with 394.46 Da. Meanwhile, it is a kind of white crystalline powder, which can considerably dissolve in ethanol and other organic solvents [13]. Therefore, brucine is a promising drug which can be used for transdermal delivery system. In the study, a novel brucine gel transdermal delivery system was developed. The therapeutical effects for anti-inflammatory and analgesia were evaluated by the adjuvant arthritis (AA) model experiment, the mouse ear swelling test (MEST) and the formalin test.

## 2. Results and Discussion

### 2.1. Adjuvant-Induced Arthritis

**Figure 1:**
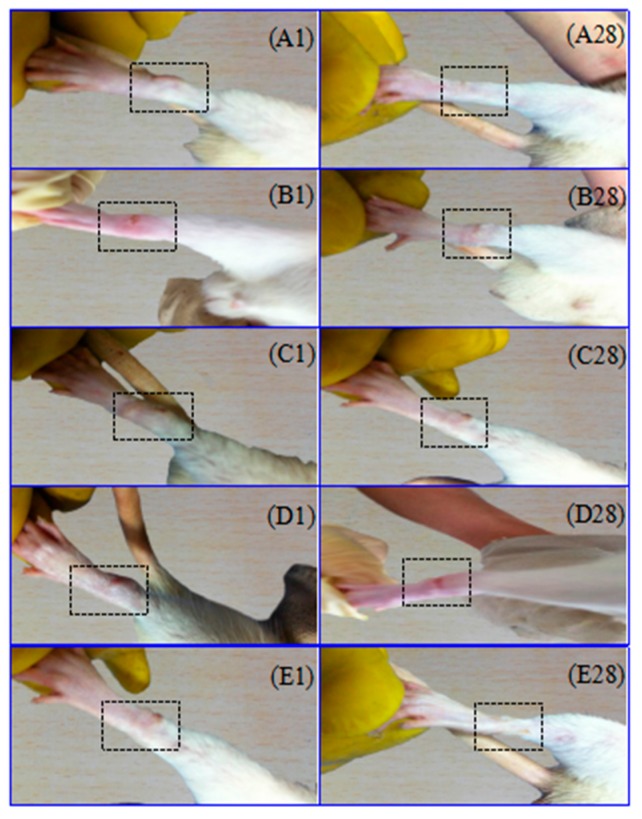
Effects of brucine gel on primary arthritis in adjuvant arthritis (AA) rats. Morphological representations of rat paw indicated the change of pre- and post-treatment in brucine gel high-dose (**A**); brucine gel low-dose (**B**); Yunnan Baiyao powder (**C**); model group (**D**); and blank control group (**E**). Images showed that the paw swelling after injection with Freund’s adjuvant complete (FAC) in the right hind paw on the first day in (**A1**,**B1**,**C1**,**D1**,**E1**). The paw swelling subsided after brucine gel and Yunnan Baiyao powder were treated respectively on the 28th day in (**A28**,**B28**,**C28**,**D28**,**E28**).

**Figure 2:**
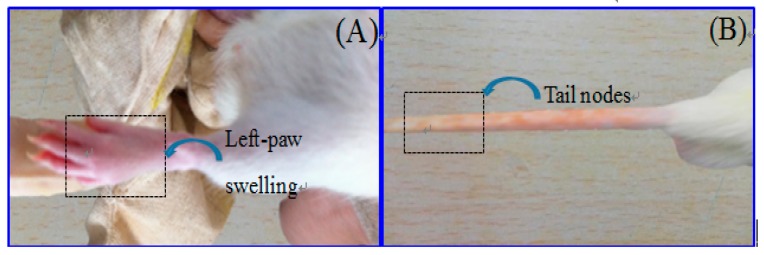
Effects of brucine gel on secondary arthritis in AA rats. Images show secondary adjuvant arthritis symptoms in (**A**,**B**), which appeared on the 13th day after injection with FAC.

All experimental animals showed some symptoms after injection with Freund’s adjuvant complete (FAC) in rat sub-plantar. The primary arthritis symptoms were that swelling and erythema of soft tissue emerged in the injected paws (Figure 1). The secondary arthritis symptoms appeared on the 13th day after injection with FAC. Its manifestations were tail nodes and non-injection site swelling, such as the left paw swelling (Figure 2). The brucine gel has been shown to reduce joint swelling and distortion, inhibit the development of arthritis, and alleviate pain and inflammation. The therapeutic effect of high doses was better than that of low doses, and similar to that of Yunnan Baiyao powder.

The model group showed significant weight loss on day 7, 14, 21 and 28 (*p* < 0.05) compared with blank control group. Compared with the model group, treatment groups of Yunnan Baiyao powder and brucine gel high-dose had significantly changed in body weight (BW) on day 7 and 14. The growth of BW became slow in all AA model rats (Table 1).

**Figure 3:**
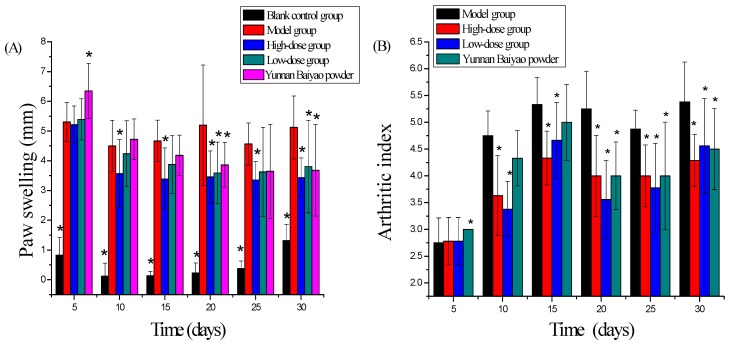
Effect of brucine gel on paw swelling (**A**) and arthritic index (**B**). Symbols represent statistical significance: * (*p* < 0.05) when compared with model group.

Paw swelling was an objective indicator for evaluating the severity of inflammation in the AA model. With the prolonged time, paw swelling became much more severe after injection with FAC. Paw swelling volume increased and reached to its maximum on the 20th day in the model group, which varies significantly compared with the blank control group. It showed that AA model was successfully established. All treatment groups showed a significant inhibition on paw swelling in comparison to the model group on the 20th, 30th (low-dose group), 10th–30th (high-dose group) and 5th, 20th and 30th days (Yunnan Baiyao powder group) respectively. Images showed the remarkable reduction of paw thickness after treatment in Figure 3A.

The arthritic index represented the grade of arthritis. It was used to assess the efficacy of brucine gel. Model group showed an increased arthritic index starting from the 10th day, and hitting the roof on the 20th day (Figure 3B). Compared with model group, arthritis scores had obviously reduced in brucine gel groups (10th–30th day) and in Yunnan Baiyao powder group (20th–30th day).

### 2.2. Synoviocytes

**Figure 4:**
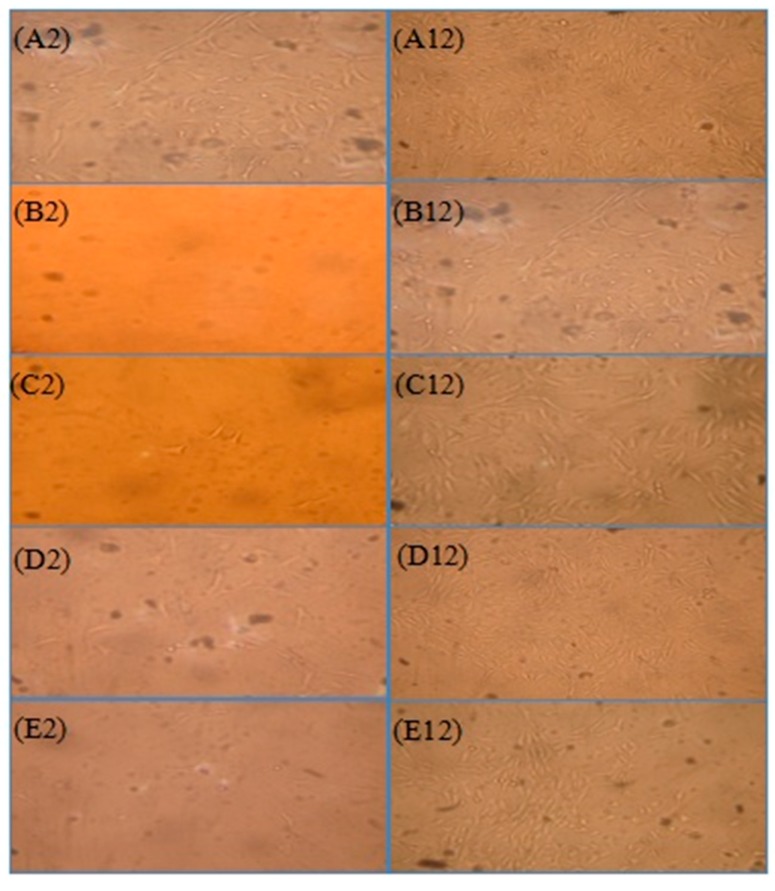
Effects of brucine gel on synoviocytes. Morphology of synoviocytes (magnification = 200×) in model group (**A2**,**A12**), blank control group (**B2**,**B12**), brucine gel high-dose group (**C2**,**C12**), brucine gel low-dose group (**D2**,**D12**) and Yunnan Baiyao powder group (**E2**,**E12**). Synoviocytes were isolated and cultured on the 2nd day (**A2**,**B2**,**C2**,**D2**,**E2**), and on the 12th day (**A12**,**B12**,**C12**,**D12**,**E12**).

The predominant shape of synoviocytes was polygon or spindle-shape fibroblast-like cells. Cytoplasm was homogeneous, transparent and uniform. Synoviocytes could proliferate steadily in vitro. The quantity of synoviocytes was high in the model group compared with other groups, which was correlated with the hyper-proliferation of synovial tissues in rheumatoid arthritis. The proliferation of synoviocytes had obvious inhibition in high-dose brucine gel group and Yun’nan White Medicinal Powder Spray group. The quantity of synoviocytes was compared among groups and was sorted: B < C < E < D < A (Figure 4).

**Figure 5:**
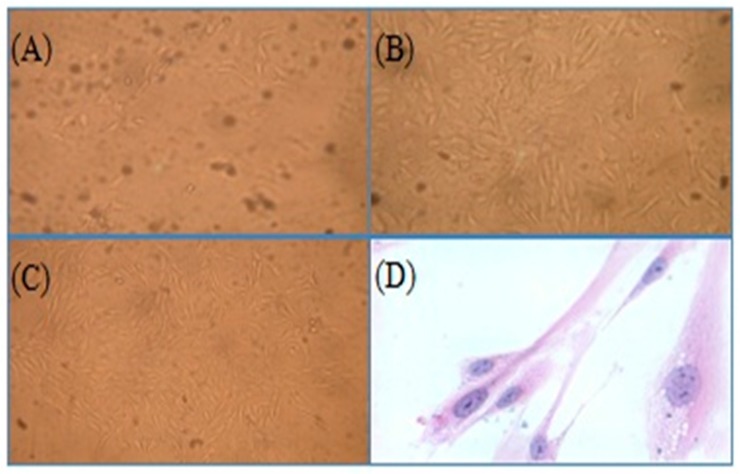
Synoviocyte purification and identification (magnification = 200×). Before purification (**A**); the first passage of type B synoviocytes (**B**); the purified synoviocytes (**C**) and the stained synoviocytes (**D**).

Synovial tissues were digested with Trypsin-EDTA to obtain the first and second passage of synoviocytes (Figure 5A,B). The number of synoviocytes increased, and the impurity reduced after purification (Figure 5C). Synoviocytes were stained with giemsa. The cytoplasm was prunosus or deep purple, and nuclei were blue, which showed that the purified cells were achieved (Figure 5D).

### 2.3. Inflammation and Soreness

**Figure 6:**
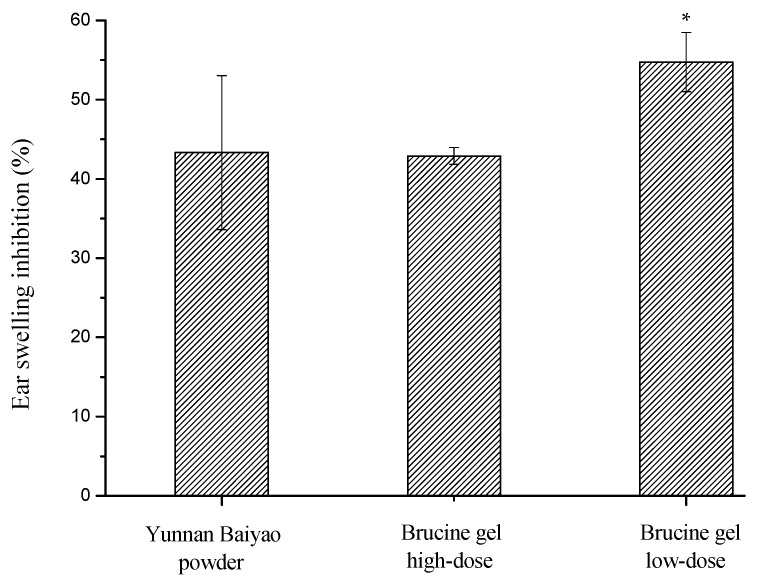
Effect of brucine gel on ear swelling. Symbols represent statistical significance: * (*p* < 0.05) compared with control group.

The xylene-induced ear edema was significantly inhibited in the high-dose group of brucine gel, the maximum inhibition rate reaching as high as 58.5% (the ear swelling was 3.57 mg). Meanwhile, the low-dose group of brucine gel and Yunnan Baiyao powder group displayed weak anti-inflammatory activities compared with the control group (Figure 6).

**Figure 7:**
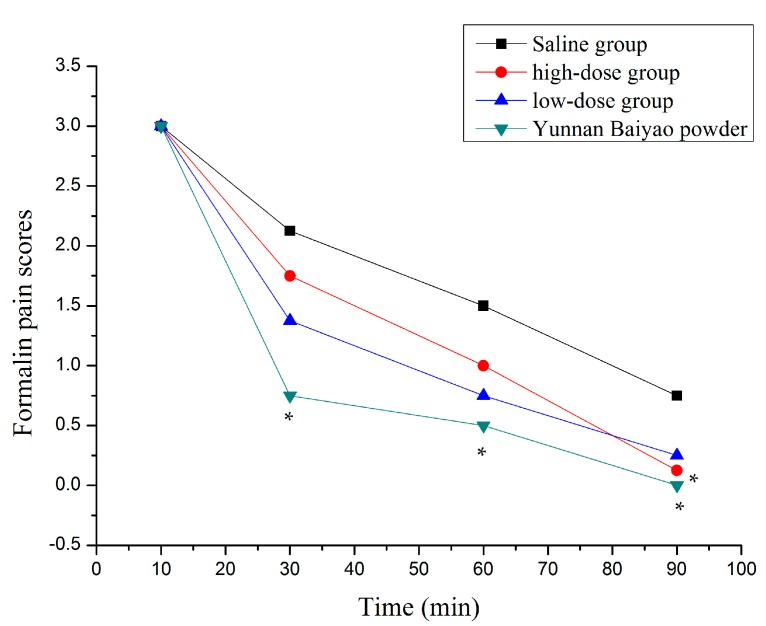
Time-effect curves of formalin pain scores (* *p* < 0.05 compared with saline group).

There were several symptoms in all experimental animals in which the injected paws were licked and not in contact with any surface, which resulted in the successful establishment of formalin-induced nociceptive model. The saline group’s pain index is higher than the other groups from 30 to 90 min (Figure 7). The Yunnan Baiyao powder group indicated significant abirritation throughout the experiment. There were statistical differences for the high dose group of brucine gel at 90 min.

### 2.4. Cytotoxicity Assay

**Figure 8:**
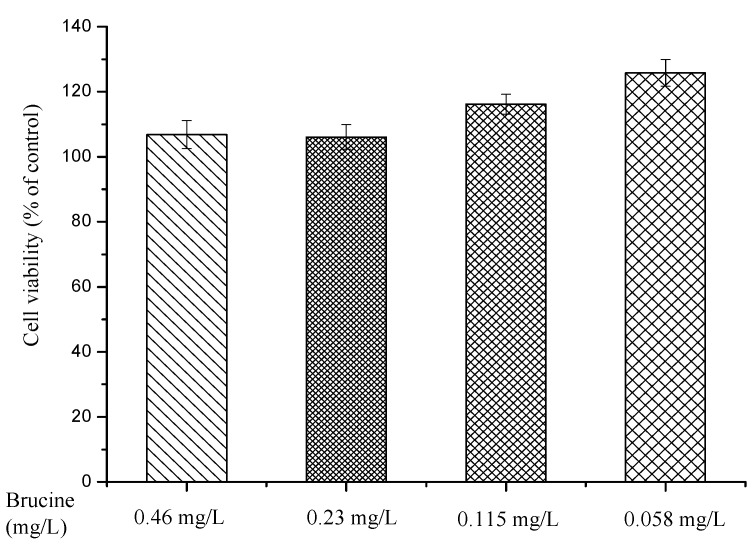
Cytotoxicity evaluation of brucine at different concentrations.

Cell viability results from Cell Counting Kit-8 (CCK-8) assay demonstrated the good security and biocompatibility of brucine (Figure 8). The RAW264.7 cells remained levels of viable cell and proliferated throughout the duration of the culture period under the action of brucine. No significant cytotoxicity was found for brucine at any of the concentrations tested. The cell viability was still greater than 113% when the concentration of brucine reached up to 0.46 mg/L.

### 2.5. PGE2 Production Assay

**Figure 9:**
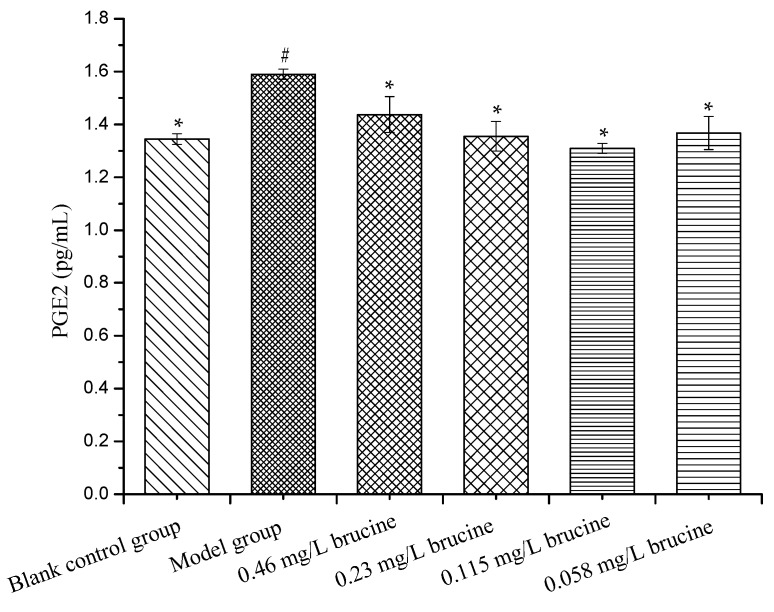
Effects of brucine on Prostaglandin E2 (PGE2) production. Symbols represent statistical significance: # (*p* < 0.05) when compared with blank control group and * (*p* < 0.05) when compared with model group.

Prostaglandin E2 (PGE2) production of RAW264.7 cells in the model group (non-treated and stimulated with LPS control cultures) showed significant difference when compared with the blank control group (non-treated control cultures). The PGE2 levels were significantly decreased after 24 h treatments with different concentrations of brucine (Figure 9). It indicated that brucine effectively inhibited LPS-induced Prostaglandin (PGE) production, without affecting cell viability.

Inflammation was a defensive biological response of the body to various injuries, which was characterized by redness, pain, swelling, heat and loss of function at the site of injury [14,15]. At the same time, the site of injury would release many pro-inflammatory mediators along with leakage of fluid from the vascular tissues [16]. In addition, the permeability of the vessel walls would be increased, and the inflammatory cells would be migrated [17,18]. All types of pain, whether it was acute or chronic, peripheral or central, initiated from inflammation [19].

Rheumatoid arthritis (RA) belongs to autoimmune inflammatory diseases affecting all age groups [20,21]. The form of RA was the persistent infection of articular surface or retention of microbial products in the synovial tissue, which generated a chronic inflammatory response [22]. Adjuvant-induced arthritis in rats was a useful tool to study the pathophysiology of RA, especially since the experimental model and the human disease shared various signs and symptoms [23]. It was demonstrated that various mediators were released by injection of FAC in the rat paw. In addition, the inception phase of inflammatory reaction might be owing to the release of histamine, but the second phase was due to the release of prostaglandin (PGs), which results in inflammatory response [24]. In the study, the results on paw swelling and arthritic index suggested that the brucine gel had a selective effect on these mediators. Their activities might be related to PGs synthesis inhibition. Synoviocytes were isolated from synovial tissues of each group after AA model experience, and evidently, the results of cell culture also showed that the synoviocytes in brucine gel group were less than in the model group. This provided further support that the brucine gel possessed anti-inflammatory activity by transdermal administration.

Mouse ear swelling test was a suitable experimental animal model for evaluation of anti-inflammatory effect. The mouse’s ear was accompanied with swelling by the release of pro-inflammatory substances, which increased capillary permeability and inflammatory cells infiltration [25]. In the research, the mice ear swelling was significantly inhibited by the brucine gel. It suggested that the brucine gel might restrain the release of pro-inflammatory substances in inflammatory tissue and reduce xylene-induced vascular permeability. In addition, brucine could permeate through the skin layer and the absolute bioavailability of transdermal administration was 32.8% in mice in the present report [10]. In the study, the high-dose group of the brucine gel exhibited markedly anti-inflammatory activity. It revealed significant effects against chemical stimuli by transdermal administration.

Formalin test was used to evaluate the analgesic effect of the brucine gel [26]. It was shown that the earlier phase of formalin-induced pain reflected formalin direct irritation to mice in the recent studies. This was due to the chemicals stimulating pain-sensing nerves [27]. The late phase reflected inflammatory pain. It was due to synthetic PGs, which decreased pain threshold and had a marked after-effect [28,29,30,31]. In this study, the brucine gel showed significant analgesic activity in the late phase, which suggested that the analgesic effect of brucine gel might be related to inhibition of the synthetic PGs.

The RAW264.7 cell line was a suitable macrophage model used to study inflammatory responses. The vitro anti-inflammatory experiments were performed to elucidate the mechanism on the anti-inflammatory and analgesic effects of brucine. The PGE2 level was markedly increased for response to LPS, and brucine significantly decreased the release of PGE2 production at any of the concentrations tested. In addition, cytotoxic effect of brucine was evaluated, and it did not affect the cell viability at all concentrations used (0.058–0.46 mg/L). These findings indicate that brucine could suppress PGE2 production in LPS-stimulated RAW264.7 cell line.

## 3. Experimental Section

### 3.1. Drugs and Animals

Freund’s adjuvant complete (FAC) and lipopolysaccharide (LPS) were obtained from Sigma Aldrich (St. Louis, MO, USA). Fetal bovine serum (FBS) was procured from gibco BRL Life Technologies Ltd. (Paisley, UK). Dulbecco’s modified Eagle’s medium (DMEM) and dimethyl sulfoxide (DMSO) were purchased from Hyclone (Logan, UT, USA), 5% Formalin physiological saline solution was purchased from Xilong Scientific Co., Ltd. (Guangzhou, China), Yunnan Baiyao powder was purchased from Yunnan Baiyao Co., Ltd. (Kunming, China), Prostaglandin E2 (PGE2) ELISA Kit was purchased from Cusabio Biotech Co., Ltd. (Wuhan, China). Cell Counting Kit-8 (CCK-8) was purchased from Beyotime Institute of Biotechnology (Haimen, China). The murine macrophage cell line RAW264.7 was obtained from the Cell Bank of Type Culture Collection of the Chinese Academy of Sciences (Shanghai, China). All other reagents were bought from solarbio science Co., Ltd. (Beijing, China).

Eighty Kunming male mice (weighing 18–20 g, *n* = 10 for each group) and 50 male wistar rats (weighing 180–220 g, *n* = 10 for each group) were purchased from the Department of Laboratory Animals, Central South University, China. The animals were fed with standard food and water ad libitum at a controlled temperature of 22 ± 2 °C and humidity of 40–70% on a 12 h light/dark cycle. All animals were acclimatized to the conditions one week before the experiments. All experiments were approved by Xiangtan University Animal Care and Use Committee and were guided for the Care and Use of Laboratory Animals. Animal qualified number was SCXK (Xiang) 2015-0017.

### 3.2. Brucine Gel Transdermal Delivery System

**Figure 10:**
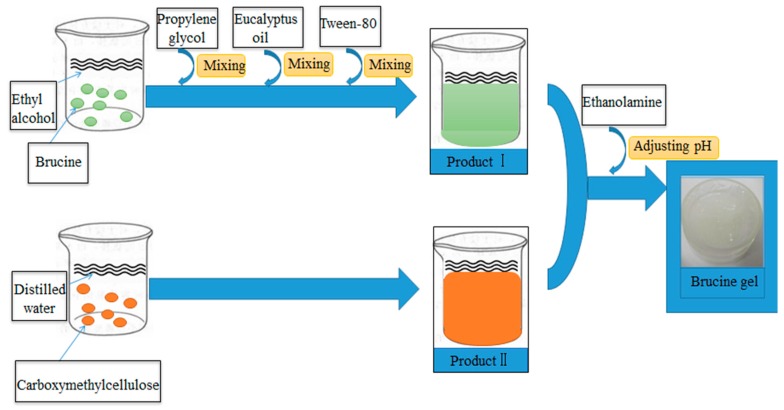
The fabrication process of brucine gel.

Brucine gel transdermal delivery system was made from carboxymethylcellulose, absolute ethyl alcohol, glycerinum, tween-80, eucalyptus oil and propylene glycol. In this pharmaceutical formulation, carboxymethylcellulose, absolute ethyl alcohol, glycerinum and tween-80 were used as a matrix, solvent, humectant and solubilizer, respectively. In addition, eucalyptus oil and propylene glycol served as penetration enhancers. Meanwhile, eucalyptus oil was also used as a preservative. The fabrication process of brucine gel was as follows (Figure 10). The brucine gel exhibited homogeneous texture with white glossy color, slight smell, no bubbles, and easily absorbed into the skin without the greasy feeling.

### 3.3. Rat Adjuvant Arthritis Model

Wistar rats (weighing 180–220 g, *n* = 10 for each group) were divided into five groups. The hind paw and dorsal hair of rats were carefully removed (4 × 4 cm^2^) with an electric clipper. The drugs (1 mL) were smeared on the area of hair removal twice daily. Except the blank control group, arthritis was induced by a sub-plantar injection of FAC (0.1 mL/paw) in the region of the right hind paw. The drugs were smeared after the injection. Two treatment groups were smeared for brucine gel (2 and 0.5 mg/mL, respectively), one positive control group was smeared for Yunnan Baiyao powder, and one blank control group and one model group were smeared for normal saline (NS). All rats were administrated continuously for four weeks. Edema in the paw was measured with a vernier caliper at five days intervals, and evaluated from the 1st day to the 30th day. Arthritic behaviors were assessed by the creation of arthritic index immediately after FAC immunization and continued for 30 days. Each rat was subjectively graded as follows [30]: 0 = absence of arthritis, 1 = tail nodules and mild swelling and/or erythema of ear, nose and paw, 2 = moderate swelling and erythema of both tarsus and ankle, 3 = severe ankylosis and bony deformity. The scores of arthritis behaviors for each at five day intervals were calculated as the weighted average of the number in each behavior. Body weight (BW) was measured on day 0, 7, 14, 21 and 28 after injection.

### 3.4. Isolation and Culture of Synoviocytes 

Synoviocytes were extracted from the experimental animals. Synovial tissues were derived from the knee joints of the wistar rats. The synovial tissues were then washed gently three times in D-HANKS (to remove the blood). After that, they were isolated by the two-digestion method [32] as following: the synovial tissue blocks were cut into small fragments, digested and stirred with collagenase in DMEM medium containing 15% FBS, 50 U/mL penicillin and 50 µg/mL streptomycin at 37 °C in an atmosphere of 5% CO_2_ for 3 h. The synovial tissue lysate was centrifuged at 1000× *g* for 10 min, then washed and digested extensively with Trypsin-EDTA. These cells were re-suspended in the culture medium which was replaced the next day.

Synoviocytes were purified by the differential attachment method. When the cells reached 80–90% confluence, they were sub-cultivated using trypsin and then cultured for 4h. After repeated attachment and passage, 3~5 passages synoviocytes gradually were purified. The purified synoviocytes were assessed by giemsa staining and confirmed by cell morphology.

### 3.5. Mouse Ear Swelling Test

Anti-inflammatory activity was evaluated using MEST. The dorsal hair was carefully removed (2 × 2 cm^2^) before the experiment. 40 Kunming mice were randomly divided into four groups (*n* = 10 for each group) and were pre-treated with the NS, Yunnan Baiyao powder, high-dose and low-dose brucine gel (2 and 0.5 mg/mL, respectively) twice a day, 0.5 mL/time. 30 min later, 0.2 mL xylene was evenly smeared on the two sides of the right ear, while the left ear was used as a blank control. One hour after xylene application, the mice were sacrificed. Both ears were cut off along edge of ear, 9 mm diameter round ear pieces were hit in the same area of both ears and then weighed accurately. Ear swelling rate was determined by the following formula:

% swelling rate = (*A − B*)/*B* × 100

where *A* and *B* were weight of the control group and the drug groups, respectively.

### 3.6. Formalin Test

The grouping and administration were carried out as previously described. The hair of dorsal area was carefully removed (2 × 2 cm^2^), the mice were smeared with the drug (0.5 mL) twice a day. Formalin solution (20 µL, 5%) was injected subcutaneously into the plantar surface of the left hind paw after 30 min treatment. Subsequently, these animals were returned to the observation cages and commenced recording the behaviours at 10, 30, 60, and 90 min. Pain behaviors were scored as follows [33]: 0 = the injected paw was not favored, 1 = the injected paw had little or no weight placed on it, 2 = the injected paw was elevated and not in contact with any surface, and 3 = the injected paw was licked or bitten.

### 3.7. Cytotoxicity Assay

The murine macrophage cell line (RAW264.7) was cultured in DMEM high glucose supplemented with 10% heat-inactivated FBS in 5% CO_2_, 95% air and humidified atmosphere at 37 °C. Cell cytotoxicity was evaluated by a modified CCK-8 assay. The cells were seeded at 1 × 10^6^ cells/mL in 96-well plate for 12 h. After attachment, the cells were treated with different concentrations of brucine (0.46, 0.23, 0.115, 0.058 mg/L) for 24 h. The medium was then discarded, and 100 µL of DMEM medium containing 10 µL of CCK-8 dye was added to each well for 2 h at 37 °C. The absorbency was measured at 450 nm (A450) with a microplate reader (Themo, Waltham, MA, USA). The A450 in control (untreated) cells was taken as 100% of viability. Five replicate wells were used for each analysis, and the experiments were repeated three times independently. Results were represented as mean ± SD.

### 3.8. PGE2 Production Assay

The RAW264.7 cells (4 × 10^6^ cells/mL) were treated with brucine (0.46, 0.23, 0.115, 0.058 mg/L) for 4 h and then stimulated with LPS (1 µg/mL) for 18 h in 24-well plates. The cell culture supernatants were collected at the allotted times, and Prostaglandin E2 (PGE2) productions were measured by a PGE2 ELISA kit according to the manufacture’s protocol. The assays were performed three times. PGE2 levels were represented as mean ± SD.

### 3.9. Statistical Analysis

The results are expressed as the $\bar{x}$ ± SD. SPSS16.0 statistical software was employed for statistical analysis with Student’s *t*-test, and considered as: significant (*) *p* < 0.05.

## 4. Conclusions

In summary, brucine, extracted from *Strychnos nux-vomica* could inhibit the production of PGE2 without affecting cell viability. A novel gel transdermal delivery system containing brucine possessed the anti-inflammatory and analgesic activities. The results concluded that brucine gel could be useful for the treatment of inflammatory diseases via transdermal delivery.

## Figures and Tables

**Table 1:** Effect of brucine gel on body weight (BW).

Groups	BW (g)				
	Day 0	Day 7	Day 14	Day 21	Day 28
Blank control group	200.52 ± 12.461	221.06 ± 13.936 *	248.48 ± 20.071 *	262.04 ± 23.671	283.14 ± 21.453
Model group	196.53 ± 19.643	193.12 ± 20.343 ^#^	212.85 ± 22.778 ^#^	224.27 ± 19.413 ^#^	246.92 ± 12.95 ^#^
High-dose group	195.15 ± 13.943	211.52 ± 15.736 *^#^	238.71 ± 27.822 *	241.44 ± 30.686	250.90 ± 35.31 ^#^
Low-dose group	202.11 ± 20.067	208.52 ± 14.293 *	227.48 ± 19.924	242.25 ± 22.407	255.21 ± 25.150
Yunnan Baiyao powder group	198.67 ± 16.918	210.08 ± 20.560 *	228.98 ± 24.936	233.12 ± 26.119	234.08 ± 32.79 ^#^

Symbols represent statistical significance: ^#^ (*p* < 0.05) when compared with blank control group and * (*p* < 0.05) when compared with model group.
